# Microbial enzymes that oxidize hydrocarbons

**DOI:** 10.3389/fmicb.2013.00338

**Published:** 2013-11-13

**Authors:** Rachel N. Austin, Amy V. Callaghan

**Affiliations:** ^1^Department of Chemistry, Bates CollegeLewiston, ME, USA; ^2^Department of Microbiology and Plant Biology, University of OklahomaNorman, OK, USA

**Keywords:** alkane oxidation, anaerobic alkane oxidation, hydrocarbon metabolism, anaerobic hydrocarbon metabolism, deepwater horizon, deep sea vents

Hydrocarbons are ubiquitous compounds of both natural and anthropogenic origin. Natural sources are derived via biosynthesis reactions in bacteria, phytoplankton, plants and fungi (Ladygina et al., 2006), as well as diagenesis and catagenesis (Horsfield and Rullkotter, 1994). As a result, hydrocarbons are environmentally distributed in microbial mats (Green and Jahnke, 2010), deep subsurface oil reservoirs (Sephton and Hazen, 2013), coalbeds (Strąpoć et al., 2011), and natural oil seeps (Kvenvolden and Cooper, 2003). Hydrocarbons are energy-rich biological substrates. However, accessing this energy requires novel enzymes. The metabolic ability of microorganisms to transform hydrocarbons is not surprising given that microbes have been exposed to these compounds throughout the course of evolution. The coupling of microbial processes and hydrocarbon transformation have important implications with regard to our understanding of biogeochemical cycling, biotransformation of environmental pollutants, subsurface production and oxidation of methane, and host/pathogen relationships. This special issue highlights recent advances in our understanding of the enzymes that govern hydrocarbon transformation in both microbial and environmental systems.

From a microbe's metabolic perspective, the largest thermodynamic benefit of hydrocarbon metabolism is derived from the oxidation of hydrocarbons with molecular oxygen. Harnessing the free energy inherent in oxygen reactivity requires an organism to have evolved mechanisms of activating oxygen and processing the reactive intermediates in ways that lead to the selective oxidation of the desired substrate, without incurring indiscriminate reactions within the organism itself. To date, the mechanisms involved in aerobic hydrocarbon oxidation are well-described, and the most prevalent feature of these processes is the dual role of oxygen as a physiological requirement and as a reactant (Berthe-Corti and Fetzner, 2002; National Research Council, 2003). Several articles in this issue review these aerobic mechanisms and the requisite enzymes. Specifically, these articles address aerobic enzymes involved in alkane oxidation (Ji et al., 2013; Wang and Shao, 2013) and new insights to the characterization of the model methanotroph, *Methylosinus trichosporium* OB3b (Matsen et al., 2013; Yang et al., 2013).

In the absence of oxygen, anaerobic and facultative microorganisms use alternative electrons acceptors, such as sulfate, nitrate, iron, manganese, and carbon dioxide to oxidize hydrocarbons. The last 30 years have yielded new insights into the novel biochemistry of anaerobic microorganisms with respect to hydrocarbon activation and degradation. Several pathways have emerged as common themes among physiologically diverse microorganisms utilizing a range of hydrocarbon substrates under a myriad of terminal electron accepting conditions (Heider and Schühle, 2013). Among recent discoveries are new mechanisms and enzymes involved in anaerobic alkane oxidation. In this issue, we have included review articles by Callaghan (2013) and Agrawal and Gieg (2013) outlining the current state of knowledge regarding anaerobic methane and non-methane alkane oxidation and methods for the *in situ* detection of anaerobic alkane biodegradation.

In addition to hydrocarbon metabolism, which is widespread among microbial taxa, some microorganisms synthesize hydrocarbons, and the purpose of these biosynthesized hydrocarbons is not well-elucidated. For example, the microalga *Botryococcus braunii* can synthesize up to 75% of its dry weight in alkanes (Banerjee et al., 2002). In some organisms, the alkanes constitute protective coatings, whereas in other microbes, they may serve as a strategy to store excess energy when other required nutrients are not available for metabolism and protein synthesis. Protective coatings, however, can serve as substrates for antagonistic microorganisms, and the strategies employed to oxidize hydrocarbons on the outside of cells appear to be different than those employed to utilize hydrocarbon stores within cells. The article by Pedrini et al. (2013) provides detailed evidence for microbial oxidation of alkanes in a host/pathogen relationship.

Given the tragedy of the Deepwater Horizon oil spill, the role of microorganisms in mitigating natural and anthropogenic hydrocarbon inputs to marine and terrestrial ecosystems has been brought to the forefront of scientific inquiry. Three papers in this issue focus on microbial activity in oil-impacted environments. Porter and Young (2013) investigate the distribution and potential use of *bamA* as a biomarker for anaerobic aromatic hydrocarbon degradation in environmental samples. Kimes et al. (2013) evaluate the use of metabolite profiling coupled with metagenomics to investigate the response of sediment microbial communities to the Deepwater Horizon oil spill. Finally, Bertrand et al. (2013) elucidate the identity and mechanisms of alkane hydroxylases found in organisms isolated from deep-sea vents, an environment in which the natural release of chemosynthetic alkanes may serve as an important fuel source.

While the flux of hydrocarbons through anthropogenic combustion is much larger than the flux via microbial processing and tends to dominate the public's attention due to concerns about global climate change, the selectivity and diversity of microbial transformations of hydrocarbons remains a powerful reminder of the unrivaled elegance of molecular evolution. The future, in which we continue to isolate novel organisms, harness significant advances in sequencing technology, and develop new environmental monitoring tools will undoubtedly unveil exciting and unexpected aspects of the fundamental roles that microbial metabolism plays in hydrocarbon transformation.
